# Donor-Specific Anti-HLA Antibodies in Allogeneic Hematopoietic Stem Cell Transplantation

**DOI:** 10.3389/fimmu.2016.00307

**Published:** 2016-08-12

**Authors:** Sarah Morin-Zorman, Pascale Loiseau, Jean-Luc Taupin, Sophie Caillat-Zucman

**Affiliations:** ^1^Laboratoire d’Immunologie et Histocompatibilité, Hôpital Saint-Louis, Assistance Publique Hôpitaux de Paris (APHP), Université Paris Diderot, Paris, France

**Keywords:** donor-specific antibodies, HLA antigens, allogeneic hematopoietic stem cell transplantation, graft failure, single antigen flow bead assay

## Abstract

Allogeneic hematopoietic stem cell transplantation (AHSCT) is a curative treatment for a wide variety of hematological diseases. In 30% of the cases, a geno-identical donor is available. Any other situation displays some level of human leukocyte antigen (HLA) incompatibility between donor and recipient. Deleterious effects of anti-HLA immunization have long been recognized in solid organ transplant recipients. More recently, anti-HLA immunization was shown to increase the risk of primary graft failure (PGF), a severe complication of AHSCT that occurs in 3–4% of matched unrelated donor transplantation and up to 15% in cord blood transplantation and T-cell depleted haplo-identical stem cell transplantation. Rates of PGF in patients with DSA were reported to be between 24 and 83% with the highest rates in haplo-identical and cord blood transplantation recipients. This led to the recommendation of anti-HLA antibody screening to detect donor-specific antibodies (DSA) in recipients prior to AHSCT. In this review, we highlight the role of anti-HLA antibodies in AHSCT and the mechanisms that may lead to PGF in patients with DSA, and discuss current issues in the field.

## Introduction

Allogeneic hematopoietic stem cell transplantation (AHSCT) is a life-saving procedure for various hematological and immunological conditions. Nowadays ~30% of AHSCT procedures are performed with human leukocyte antigen (HLA)-identical siblings. Any other situation displays some level of HLA incompatibility between donor and recipient, even in 10/10 matched unrelated donors where a mismatch for HLA-DPB1 and/or DRB3, B4, or B5 is present in 80% of the cases. Deleterious effects of anti-HLA immunization have long been recognized in solid organ transplantation (1–3). More recently, anti-HLA immunization was shown to increase the risk of graft failure in AHSCT. For this reason, it is now strongly recommended to screen recipients for anti-HLA donor-specific antibodies (DSA) before AHSCT. In this review, we outline the role of anti-HLA antibodies in AHSCT and discuss current issues in the field.

## Techniques for HLA Antibody Detection and Identification

Complement-dependent lymphocytotoxicity (CDC) tests that were used initially have been widely replaced by bead-based assays, such as the single antigen flow bead (SAFB) assays that are more sensitive and resolutive (4). This technique allows antigen-specific and even allele-specific antibodies to be identified, using fluorescent micro-spheres each coated with a unique HLA variant/allele. Each bead subpopulation has a unique color resulting from the mixture of two fluorochromes, all the beads from the same class being mixed together in a multiplex approach. Currently, a panel of nearly 100 class I alleles and quite as many for class II, can be analyzed with minute serum amounts in less than 2 h. Serum is incubated with the beads, unbound serum components are removed through washing, and a phycoerythrin-labeled anti-human IgG is added to identify the HLA alleles recognized by serum antibodies thanks to bead fluorescent coding. Reactivity is measured by Luminex-based technology, using a software program for subtracting background fluorescence of a negative control serum, and is reported as a mean fluorescence intensity (MFI) value. This technique is much more sensitive than the CDC approach, and slightly more than the classical cell-based flow cytometry assay. It, therefore, allows very weak antibodies to be identified, which were not detectable before. This method measures antibody strength, resulting from the concentration of the antibody in the serum combined with antibody affinity/avidity for the antigen. After nearly 10 years of use, it is still not clear in organ transplantation what the clinically relevant threshold is, as a deleterious clinical impact has been reported at very low MFI (in the 500 range) for kidney transplantation (5). This technique has other limitations, besides its high costs: false-positive reactions possibly caused by co-purified irrelevant antigens, or high level of background reactivity in some patients. Another potential issue is complement interference, the so-called “prozone” effect, that is due to downstream C4- and C3-derived complement activation products that mask the recognition site of the PE-labeled anti-human IgG conjugate and impairs its binding. This can lead to underestimating anti-HLA antibody strength, especially for the strongest ones that would need to be at least detected, if not correctly estimated through their MFI levels. This phenomenon is avoided by preventing complement activation though pre-treatment of serum with EDTA, DTT, or heat, which knock down the complement classical pathway at the initial step involving the calcium-dependent C1 complex. Despite these downsides, SAFB assays have revolutionized the field, by allowing the precise identification of antigenic targets for anti-HLA antibodies, a goal that could not be technically reached before their advent.

## Origin and Prevalence of Anti-HLA Antibodies

“Natural” anti-HLA antibodies can exist in healthy, non-transplanted, non-transfused, non-parous individuals, at a prevalence estimated to be between <1 and 5% (6, 7). Some “natural” antibodies are reactive against denatured or cryptic HLA epitopes that are normally hidden in the well-conformed native molecule, whereas others are reactive against native epitopes. Natural antibodies are considered as resulting from cross-reactions with common environmental antigens encountered by individuals all along their lives. Antibodies reactive against denatured epitopes interact with HLA molecules that are ill-configured because of natural instability or due to procedures used to produce, isolate, and adsorb the antigen on the beads (8). Of note, some studies reported on a much higher rate of this kind of allo-immunization, reaching up to 63% in non-parous individuals (9). This discrepancy may be explained by different techniques used in these studies and/or variable cut-off values for positivity or different bead engineering (10).

Besides natural antibodies, main causes of anti-HLA antibody development are pregnancy, blood product transfusion, and previous transplantation. The incidence of anti-HLA immunization through platelet transfusion was strongly decreased by leukocyte depletion of blood products: in a large trial, it plummeted from 45% in controls to 17% in non-parous patients who received leukocyte-depleted platelets (11). However, leukocyte reduction did not solve the issue of allo-immunization related to red blood cell transfusion. The rate of allo-immunization in transfused non-parous individuals who received leukocyte-depleted red blood cells was not reduced in three randomized control trials (12–14): the allo-immunization rate ranged from 10% in low-risk patients to over 50% in females with prior pregnancy. In patients suffering from hematological diseases, anti-HLA immunization ranges from 19.6 to 39.4% (Table 1).

**Table 1:** **Studies of DSA impact in different settings in AHSCT**.

Reference	Patients (*n*)	Stem cell source	Conditioning	Anti-HLA%	DSA%	Graft failure with/without DSA
Spellman et al. (34)	115	Mismatched unrelated	RIC	ND	9	24 versus 1%
Ciurea et al. (36)	592	10/10 and 9/10 unrelated	MACorRIC	19.6	1.4	37.5 versus 2.7%
Yoshihara et al. (39)	79	Haplo-identical	RIC	20.2	14	27 versus 3%
Ciurea et al. (36)	24	Haplo-identical	RIC	ND	21	60 versus 5%
Chang et al. (40)	345	Haplo-identical	MAC	25.2	11.3	61% (MFI_>_10,000) versus 3.2%
Ciurea et al. (36)	122	Haplo-identical	Non-specified	ND	18	32 versus 4%
Takanashi et al. (41)	386	Single CBU	MAC	23.1	5	83 versus 32%
Cutler et al. (42)	73	Double CBU	MACorRIC	ND	24	57 versus 5.5%
Ruggeri et al. (43)	294	Single and double CBU	RIC	23	5	81 versus 44%
Yamamoto et al. (44)	175	Single CBU	MACorRIC	39.4	ND	50% if anti-HLA-C, DP, DQ, DRB1/2/3 versus 16%

## Immunization Against Donor HLA Molecules is Associated with Primary Graft Failure in AHSCT

Studies in the early 2000s associated detection of complement-fixing antidonor antibodies in the recipient by cross-match test with an increased risk of AHSCT graft failure (15, 16). Although one case report mentioned the occurrence of graft failure related to post-transplantation DSA acquisition (17), pre-transplant DSA but not *de novo* post-transplantation DSA have been correlated to primary graft failure (PGF). PGF includes graft rejection, defined by the inability to achieve a neutrophil count of 0.5 g/l for three consecutive days at day 28 post transplantation in the absence of donor hematopoiesis. It also includes poor graft function that is a failure to achieve adequate blood counts (neutrophils >0.5 g/l, hemoglobin >8 g/dl or platelets >20 g/l) for three consecutive days in the presence of complete donor hematopoiesis (18, 19). PGF is a severe complication occurring in 3–4% of matched unrelated donor transplantation and in up to 15% of cord blood and T-cell depleted haplo-identical AHSCT (20, 21). This complication considerably increases the early non-relapse mortality after allogeneic stem cell transplantation (22–25). The mechanisms are little known since only few studies have addressed them.

## Mechanisms of Graft Failure in AHSCT

Mechanisms of alloantibody generation and effector functions have been well studied in solid organ transplantation (26). Studies that investigated the mechanisms of AHSCT graft rejection in murine models showed the dominance of humoral immunity in major histocompatibility complex (MHC) allosensitized mice. Passive transfer of serum from sensitized mice was sufficient to induce rejection in naïve recipients (27). Other authors showed that antibody-mediated rejection in primed recipients was far more rapid than T-cell-mediated rejection in non-primed recipients (28). Importantly, this study suggested that antibody-dependent cell-mediated cytotoxicity (ADCC) was the primary mechanism of rejection: allosensitized FcGR^−/−^ recipients did not reject their grafts. In human, complement activation has long been known in donor-sensitized patients in solid organ transplantation, through the historic complement-dependent cytotoxicity cross match and the deposited C4d staining in biopsies that are hallmarks of humoral rejection, and more recently through the negative impact of C1q binding (29) or C3d binding (30) DSA in SAFB assays. Whether it also represents a significant mechanism of rejection in AHSCT remains unclear. However, recently, a study showed that patients with C1q-binding DSA pre-existing before AHSCT were at higher risk for PGF (31).

The consequence on hematopoietic stem cells was demonstrated *in vitro*: CD34 + stem cells incubated in the presence of complement and anti-class I or anti-HLA-DR, but not anti-HLA-DQ antibodies, were not capable of differentiating into lineage producing colonies (32). Anti-HLA-DP antibodies were shown in another study to have a modest (30%) effect on human myeloid, erythroid or multipotential progenitors but no direct impact on CD34 + cells was demonstrated (33).

## Impact of DSA in Distinct Hematopoietic Stem Cell Transplantation Settings

Approximately 30% of patients in need for AHSCT have a HLA geno-identical donor. If not, transplantation is performed with HLA-compatible unrelated donors, or alternative sources of hematopoietic stem cells, such as HLA-incompatible unrelated donors, cord blood, and, increasingly, haplo-identical donors. Table 1 shows the frequency of pre-transplant anti-HLA and DSA in AHSCT recipients, and the consequences on graft failure, according to the stem cell source.

### Impact of DSA in the Matched Unrelated Donor Setting

In the matched unrelated donor setting in Europe, HLA typing is performed for A, B, C, DRB1, and DQB1 loci and a 10/10 or at least 9/10 match is sought for. By contrast, in the US, DQB1 typing is not required, and a compatibility of 8/8 is considered as sufficient. In both continents, HLA-DPB1 matching is not required.

In one early study on 60 patients undergoing one-mismatch intra-familial transplantation or unrelated donor transplantation, the presence of anti-HLA antibodies detected by serum cross-match technique was associated with a significantly increased risk of graft failure when the cross-match test was positive (16). In another study, the authors retrospectively studied 115 patients who had received myeloablative conditioning (MAC) with at least one mismatch among A, B, C, DRB1, DQB1, or DPB1 loci (34). When comparing the frequency of pre-graft DSA in the no-engraftment group versus the engraftment group, they found that 24% of patients who did not engraft had DSA pre-transplant versus 1% in patients who did engraft.

To date, the importance of anti-HLA-DPB1 immunization is still debated, especially since DPB1 compatibility is not considered when allocating transplants. As a consequence, 80% of transplants are performed across a HLA-DPB1 mismatch (35). In a prospective study, the authors studied 592 recipients of unrelated transplants (85% of 10/10 and 16% of 9/10 matched transplants) who received MAC. DSA were present in 1.4% of patients, all directed against HLA-DPB1. Graft failure occurred in 3.2% of cases. This event occurred in 2.7% of patients without DSA compared with 37% of patients with DSA (36). Very little is known about the impact of DP expression levels on hematopoietic stem cells on risk of PGF. Interestingly, in this study, HLA DP expression was significantly lower on CD34^+^ from peripheral blood than from BM, suggesting that risk of PGF might be less important when grafts are obtained from peripheral blood. However, in a retrospective study on 2716 patients who received matched unrelated and mismatched unrelated AHSCT, the engraftment rate was not different in patients receiving stem cells from BM versus peripheral blood (3.7 and 3.9% of graft failure, respectively, *p* = 0.83) (37).

### Impact of DSA in the Haplo-Identical Transplantation Setting

In haplo-identical transplantation, only one gene for each locus is matched between donor and recipient. This procedure is undergoing a rapid development because it offers the possibility of having a donor for AHSCT to nearly every patient. Thus, it was crucial to investigate the impact of DSA in this setting. In a retrospective case-control study, which included 24 patients who received T-depleted haplo-identical transplantation with reduced intensity conditioning (RIC), the incidence of pre-transplant DSA was 21%. Risk of graft rejection was increased from 5% in the non-DSA group to 60% in the group with DSA (38). More recent studies were done in T-cell replete haplo-identical transplantation settings. The authors reported that among 79 patients transplanted with RIC, 14% had DSA, and graft failure was reported in 27% among them versus 3% in the no DSA group (39). Larger studies confirmed that the presence of DSA was associated with PGF. In a study on 345 patients with MAC conditioning, 11.3% had DSA and presence of DSA was associated with PGF in multivariate analysis (40). In a study on 122 patients, 18% of patients had DSA before transplantation and among them, 7 (32%) experienced graft failure (31).

### Impact of DSA in the Umbilical Cord Blood Transplantation Setting

In this setting, only HLA-A, -B, and -DRB1 matching is searched for, but not absolutely required. An effect on graft rejection of antibodies against these antigens when mismatched was found in a study on 386 adult patients with MAC conditioning. Importantly, this study was done with single cord blood unit (CBU), which on its own probably partly explains the high rates of graft failure (83% in group with DSA versus 32% in group without DSA) (41). In a study on 73 patients who received double CBU with mostly RIC conditioning, PGF occurred in 18% of patients who had DSA against one CBU and in 57% of patients who had DSA against both CBU versus only 5.5% in the no DSA group (42). In another study performed on 294 patients with RIC who received one or 2 CBU, engraftment rate at day 60 was 81 versus 44% in the no DSA group and TRM was also increased from 32 to 43% in patients with DSA (43). The impact of DSA against HLA-C, -DP, -DQ, and -DR3/4/5 was specifically investigated in another study on 175 patients receiving single CBU, showing that engraftment rate was inferior in HLA-mismatched patients who had anti-HLA antibodies against HLA-C, -DP, DQ, and -DRB3/4/5 compared to patients without anti-HLA antibodies or to patients who had antibodies against HLA-A, -B, or -DRB1 but no DSA. Because typing of HLA-C, -DP, -DQ, and -DRB3/4/5 loci was lacking, the authors hypothesized that unrecognized DSA were responsible for the lower engraftment rates (44).

## Impact of DSA MFI Strength

Several studies have shown that higher MFI of DSA were associated with an increased rate of graft failure (36, 38–40, 42, 43). However, there is no consensus on a clear cut-off above which the DSA is likely to cause graft failure. In a study, the authors suggested the clinically significant cut-off could be the positivity of the SAFB C1q assay (31), which is grossly correlated with antibody MFI strength in the IgG assay setting, in the field of organ transplant recipients (45–48). However, larger studies are needed to better determine a clear cut-off value.

## Role of Donor-Derived HLA Antibodies in AHSCT Recipients

Very little is known regarding the role of donor-derived anti-HLA antibodies. Donor-derived antibodies might emerge in recipients of AHSCT because the graft contains memory B cells that can differentiate into plasma cells that could be specific for a given recipient HLA determinant. One study reported the frequent detection of anti-HLA antibodies in patients who underwent AHSCT from anti-HLA antibody-positive donors (49). One group screened 127 donors for anti-HLA antibodies. Seven donors were anti-HLA antibody positive. Among the seven patients who received AHSCT from these donors, four subsequently became anti-HLA antibody positive, with antibodies that closely resembled the antibodies found in their donors. The antibodies had MFI that peaked between Days 20 and 30 in all patients, then decreased and disappeared before Day 100 (50). In a case report about two patients who received haplo-identical transplantation from anti-HLA antibody donors, the recipients became anti-HLA antibody positive and stayed positive for less than a year (51). In these studies, the antibodies were not recipient specific. The impact of recipient-specific compared to non-recipient-specific antibodies is not known. In a recent study, the authors studied a cohort of 82 HLA class II mismatched unrelated AHSCT donor-recipient pairs (52). In this cohort, 26 donors (32%) had at least one anti-HLA class II antibody detected in peripheral blood. The recipients of a graft from an anti-class II immunized donor had a significantly higher 2-year cumulative incidence of a first episode of either acute or chronic graft-versus-host disease (88 versus 67%), suggesting that donor immunization against foreign HLA antigens could be a new parameter to consider for predicting the risk of GVHD after HLA-mismatched unrelated HSCT. However, this observation needs to be confirmed on a larger cohort and the impact of recipient-specific antibodies to be studied.

## Strategies for DSA Reduction before Transplantation

The most widely used strategy in case of pre-transplant DSA is to identify and select another donor. Since this is not always possible, several reports mentioned successful strategies to reduce plasma DSA strength prior to transplantation. These reports most often describe single patients or very small series and are possibly biased since failure to reduce DSA might have remained unreported in many cases. The most frequent strategy involved B-lymphocyte depletion with rituximab and plasma exchanges (38, 39) sometimes combined with high-dose intravenous immune globulins or infusion of irradiated donor lymphocytes (31). The reduction of DSA with these treatments was variable but often only partially effective, treatment efficacy being likely related to pre-existing DSA levels. Another strategy involved transfusion of platelets bearing HLA antigens corresponding to DSA, which induced rapid and significant DSA reduction (39, 53). This strategy could be associated with rituximab in order to reduce DSA-producing B-lymphocytes (53). Lastly, bortezomib associated with dexamethasone was tested in one patient with a moderate DSA reduction (39).

There is no consensus regarding the appropriate titer at which a strategy of antibody reduction should be applied in HSCT, as there is no consensus on the clinically relevant threshold for DSA strength. There is no consensus in organ transplantation either, but recent reports strongly suggest that DSA clinical impact occur earlier as its serum strength increases at time of transplantation (29, 47) In the AHSCT setting, the situation is different, because as soon as complete donor chimerism is reached, DSA production by recipient B cells disappears. For this reason, the threshold for DSA levels that might require therapeutic intervention could be different between both settings.

In conclusion, although the mechanisms underlying the association between anti-HLA DSA and graft rejection in AHSCT are unclear, numerous studies report on a deleterious effect of DSA on AHSCT engraftment, without clearly identifying innocuous HLA loci. Further studies are needed, especially regarding the cut-off of MFI values that have clinical consequences on graft survival, and the impact of anti-DP antibodies, since mismatches are most frequent in this locus even for 10/10 matched unrelated donors.

## Author Contributions

SMZ wrote the manuscript. PL, JLT and SCZ commented on the manuscript at all stages of writing and edited it. All authors listed have made substantial, direct, and intellectual contribution to the work and approved it for publication.

## Conflict of Interest Statement

The authors declare that the research was conducted in the absence of any commercial or financial relationships that could be construed as a potential conflict of interest.
